# Benchmarking and reducing length of stay in Dutch hospitals

**DOI:** 10.1186/1472-6963-8-220

**Published:** 2008-10-24

**Authors:** Ine Borghans, Richard Heijink, Tijn Kool, Ronald J Lagoe, Gert P Westert

**Affiliations:** 1 Tranzo, Tilburg University, PO Box 90153, 5000 LE Tilburg, The Netherlands; 2 Prismant, research institute for health care, department Quality and Safety, Papendorpseweg 65, 3528 BJ Utrecht, The Netherlands; 3 RIVM National Institute for Public Health and the Environment, PO Box 1, 3720 BA Bilthoven, The Netherlands; 4 Hospital Executive Council, PO Box 35089, University Station, Syracuse, New York 13235, USA

## Abstract

**Background:**

To assess the development of and variation in lengths of stay in Dutch hospitals and to determine the potential reduction in hospital days if all Dutch hospitals would have an average length of stay equal to that of benchmark hospitals.

**Methods:**

The potential reduction was calculated using data obtained from 69 hospitals that participated in the National Medical Registration (LMR). For each hospital, the average length of stay was adjusted for differences in type of admission (clinical or day-care admission) and case mix (age, diagnosis and procedure). We calculated the number of hospital days that theoretically could be saved by (i) counting unnecessary clinical admissions as day cases whenever possible, and (ii) treating all remaining clinical patients with a length of stay equal to the benchmark (15^th ^percentile length of stay hospital).

**Results:**

The average (mean) length of stay in Dutch hospitals decreased from 14 days in 1980 to 7 days in 2006. In 2006 more than 80% of all hospitals reached an average length of stay shorter than the 15th percentile hospital in the year 2000. In 2006 the mean length of stay ranged from 5.1 to 8.7 days. If the average length of stay of the 15^th ^percentile hospital in 2006 is identified as the standard that other hospitals can achieve, a 14% reduction of hospital days can be attained. This percentage varied substantially across medical specialties. Extrapolating the potential reduction of hospital days of the 69 hospitals to all 98 Dutch hospitals yielded a total savings of 1.8 million hospital days (2006). The average length of stay in Dutch hospitals if all hospitals were able to treat their patients as the 15^th ^percentile hospital would be 6 days and the number of day cases would increase by 13%.

**Conclusion:**

Hospitals in the Netherlands vary substantially in case mix adjusted length of stay. Benchmarking – using the method presented – shows the potential for efficiency improvement which can be realized by decreasing inputs (e.g. available beds for inpatient care). Future research should focus on the effect of length of stay reduction programs on outputs such as quality of care.

## Background

**Figure 1:**
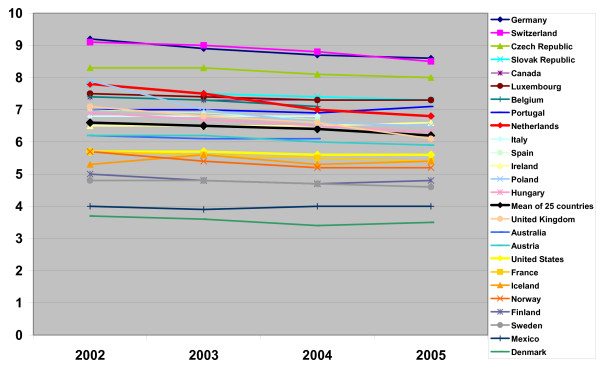
**25 OECD countries: Average length of stay in days for acute care**. In the legend countries are sorted according to the length of stay in 2005. Source: OECD HEALTH DATA 2007, July 07.

"Reducing length of hospital stay is a policy aim for many health care systems and is thought to indicate efficiency" [1]. The average length of stay of patients in Dutch hospitals has been decreasing for decades. In spite of this reduction, the length of stay in the Netherlands was longer than the combined mean length of stay of 25 OECD countries (Figure 1) during the period 2002–2005. In 2005 the mean length of stay in the Netherlands (6.8 days) exceeded the mean of the 25 OECD countries combined (6.2 days) by ten percent. Dutch lengths of stay exceeded those in the United States by 21 percent (2005). A study of the Netherlands Board for Health Facilities also showed that a further reduction of lengths of stay in Dutch hospitals might be possible [2,3].

These findings may be explainable because until 2005, the financing system in the Netherlands did not encourage length of stay reduction. Hospitals were paid through a system based, in part, on hospital patient days. Medical specialists were paid separately from this system, mostly on the basis of a lump sum. Hospitals still had several reasons to reduce length of stay. For example, the Dutch Ministry of Health Care encouraged hospitals to reduce the number of beds from 3.8 to 2.0 beds per 1000 inhabitants. Hospitals feared that their new building plans would only be accepted if they anticipated this objective to reach 2.0 beds per 1000 inhabitants[4]. Other reasons for hospitals to reduce lengths of stay included shortages of personnel and reductions in admissions caused by bedshortages. These relatively indirect incentives to reduce length of stay applied to hospitals, but not to medical specialists.

Recently, the introduction of a new financing system for hospitals, the Diagnosis Treatment Combination system (in Dutch: DBC) substantially increased the incentive for Dutch hospitals to shorten lengths of stay. This is a Dutch variation of the Diagnosis Related Group system; hospitals are paid for every DBC. At the start of the DBC-system the prices of 10% of all DBC's were negotiable between hospitals and health insurance companies. This percentage is growing. The objective is that 65–70% of all hospital care will be negotiable in 2011. For medical specialists the financing system will also change. The lump sum will be abolished and some kind of competitive system will be introduced as an intermediate phase to entirely free prices. The essence of the new financing system is to reorganize health care on a free market-basis. This new financing system gives hospitals and specialists a strong motivation to reduce costs and lengths of stay.

These developments raise the question, how many hospital days potentially could be reduced in the Netherlands in the near future? Brownell et al. (1995) determined the potential savings by reducing length of stay in eight major acute care hospitals in Manitoba [5]. Hanning (2007) benchmarked the length of stay in Australia in private cases in private facilities [6]. Both found that a substantial proportion of days could be eliminated if hospitals worked as efficiently as the benchmark.

In this study we present a method to make a realistic calculation of the potential reduction of hospital days. We will assess the development of lengths of stay in Dutch hospitals and calculate the potential reduction of length of stay if all hospitals would work as efficiently as the benchmark (the 15^th ^percentile hospital).

## Methods

### Setting: 69 hospitals

For this study, we used hospital data that were registered in the National Medical Registration (Landelijke Medische Registratie, LMR). All data were provided by research Institute Prismant. In the LMR, data are available of admissions in general and academic hospitals in the Netherlands. This information includes medical data such as diagnoses and surgical procedures as well as patient specific data, including age, gender and hospital stay. The LMR is not based on DBC's but diagnoses are classified by the ICD-9 and procedures by the Dutch Classification System of Procedures. There have been no major changes to these classification systems between 1991 and 2006.

Participation in the LMR is voluntary. Until 2004, the participation percentage of hospitals to the LMR was nearly 100%. Since 2005 some hospitals (2005: 2, 2006: 11) stopped their participation to the LMR because of the introduction of a second hospital registration: the registration of DBC's. This registration is obligatory and these hospitals gave priority to the DBC-registration instead of prejudicing the LMR-registration. Despite this diminishing number of participating hospitals we decided to use the 2006 data, the most recent available.

In 2006, the total number of general and academic hospitals in the Netherlands was 96; 11 of these hospitals did not participate in the LMR and 16 hospitals participated but did not register their procedures in the LMR. We excluded both of these groups in our analysis. Sixty nine hospitals (72% of the total) did contribute to this study. The excluded hospitals did not have a specific pattern in their lengths of stay. In 2004 their combined average length of stay was the same as the combined average length of stay of the 69 hospitals that were included in our study. For this reason we assumed that the data used in this study were representative of all Dutch hospitals.

A specialty was included if it had 100 or more clinical discharges. For eleven specialties, a number of hospitals were excluded because they produced too few discharges. The number of hospitals that were excluded varied from 57 hospitals for ophthalmology (a specialty that mainly works in outpatient clinics) to 1 hospital for orthopaedic surgery.

### Standardisation

In order to compare length of stay between hospitals we applied two adjustments:

#### 1) Adjustment for differences in the policy of admission (clinical or day-care admission)

Dutch hospitals differ in their admission policies. In principle, there is a choice between outpatient-care, day-care and clinical admission. Outpatients are treated in outpatient departments, where they consult a doctor, nurse or paramedic. Day-care is defined as care given in a specific centre for day-care to patients that only stay for several hours during the day (no overnight). Clinical patients are treated in the clinical department. They occupy a bed on a clinical ward and they intend to stay one or more overnight(s). Some hospitals tend to treat patients presenting for small procedures in day-care, while other hospitals have a larger threshold to treat in day-care. They tend to treat these patients on a clinical ward. If these patients are admitted in a clinical department, their (relatively short) length of stay contributes to the overall mean length of stay, while it does not if these patients are treated in day-care. Thus, hospitals with a larger threshold to treat patients in day-care more easily reach a short mean length of stay. In order to correct for this we excluded all hospital days of patients admitted on a clinical ward while they in principle could have been treated in day-care. In our study the hospital stay of these patients was analyzed separately. This is in accordance with the recommendation Hanning [6] made to differentiate between same-day and overnight cases in benchmarking length of stay.

Admissions that could in principle have been treated in day-care were selected on the basis of the occurrence of the main procedure in day-care. We listed all day-care procedures that were performed at least 50 times in the Netherlands in 1997 in at least 5 hospitals. Clinical admissions with a main procedure that appeared on this list were counted as admissions that could in principle have been treated in day-care if they also complied with all of the following conditions:

• Non-acute admission;

• Admission not for delivery;

• Patient did not die in hospital;

• Maximum clinical length of stay of three days;

• Only one specialty was responsible during the stay (no transfer to another specialty);

• No transfer to another hospital.

The year 1997 was used as reference to ensure that admissions really could be treated in day-care and to avoid discussions between professionals. Therefore, there is a chance for underestimation.

#### 2) Adjustment for case-mix

A valid comparison of lengths of stay requires case-mix adjustment. Therefore we computed for each hospital specialty a ratio of actual length of stay to expected length of stay. The expected length of stay was computed by Prismant. For each specialty the expected length of stay was based on the characteristics of its patients and the national mean length of stay that is associated with these characteristics[7]. A ratio higher than one indicates that the length of stay is higher than if its patients had national length of stay rates. The following characteristics (variables) were taken into account:

• Age, divided in 5 classes: 0, 1–14, 15–44, 45–64, 65+ years;

• primary diagnosis. This is the main diagnosis that led to the admission); it includes about 1,000 diagnoses classified by the ICD9 in three digits;

• procedures, classified by the Dutch Classification System of Procedures. The procedures considered depend on the diagnosis of the patient. On average it includes five procedure groups.

Together these three parameters produced about 5 × 5 × 1,000 = 25,000 cells for which the mean length of stay is taken as the expected length of stay. An exception was made for patients with a length of stay of 100 hospital days and longer and for patients who died in hospital. For the latter two groups the expected length of stay was kept equal to the actual length of stay and consequently the ratio of actual length of stay to expected length of stay always was 1.

### 15^th ^percentile hospital

In an Australian benchmark Hanning used the minimum length of stay as the standard (at state level) [6]. Brownell used the hospital with the shortest overall length of stay to calculate the potential savings [5]. For our calculation of the potential length of stay reduction, we used the 15th percentile hospital as the benchmark value. The 15^th ^percentile hospital of each specialty was determined by ranking the quotients of actual to expected length of stay of all hospitals with 100 or more discharges for each specialty. The hospital with the lowest ratio of actual to expected length of stay was identified as the hospital with the shortest length of stay. For each specialty the length of stay at the 15^th ^percentile hospital in this ranking was used as the standard for calculating the potential reduction of length of stay in all hospitals with a longer length of stay. For 2006, we calculated how many hospital days Dutch hospitals could have reduced if they had all been at least as efficient with their beds as the 15^th ^percentile hospital.

Experiences gained in our consultancy practice have shown that setting a realistic goal motivates medical specialists to reduce the length of stay. In the first years of our consultancy practice we used the minimum as the standard, but medical specialists had many problems with this approach. They continued emphasizing potential 'rest'-variation which was not standardized for. The use of the minimum as a standard discouraged them to work on improving the health care process. They saw it as an unattainable goal. By using the 15^th ^percentile and not the minimum we captured potential rest variation which was not adjusted for.

### Calculation of the potential reduction of length of stay in Dutch hospitals

To calculate the length of stay reduction that Dutch hospitals can achieve based on the results of the 15^th ^percentile hospitals, we distinguished between hospital days that could be gained by substitution from clinical to day-care and hospital days that could be gained by treating clinical patients with a shorter length of stay.

An example for internal medicine:

• In the 69 hospitals of this study the total number of hospital days in clinic and day-care was 1,467,522;

• 215,587 patients were treated in day-care and 501 were treated in clinic only for 1 day;

• 3,965 patients were admitted in clinic for a 2-day (2,867 patients) or 3-day (1,098 patients) stays but could potentially have been treated in day-care;

• Treating them in day-care would save 2,867 + 1,098 + 1,098 = 5,063 hospital days, which is 0.3% of all hospital days in clinic and day-care combined;

• Without the (potential) day-care patients the total number of hospital days was 1,242,406, generated by 139,904 patients;

• The 15th percentile hospital had a ratio of actual to expected length of stay of 0.95. Using this ratio to all expected lengths of stay of every hospital, the total gain in hospital days could be 162,868, which equalled 11.1% of all hospital days in clinic and day-care combined.

As a result, for internal medicine the hospital days that could be gained by substitution from clinical to day-care was 0.3%. Hospital days that could be gained by treating clinical patients with a shorter length of stay amounted to 11.1%. The combined level was 11.4%.

## Results

### 1) Development of length of stay in Dutch hospitals

**Figure 2:**
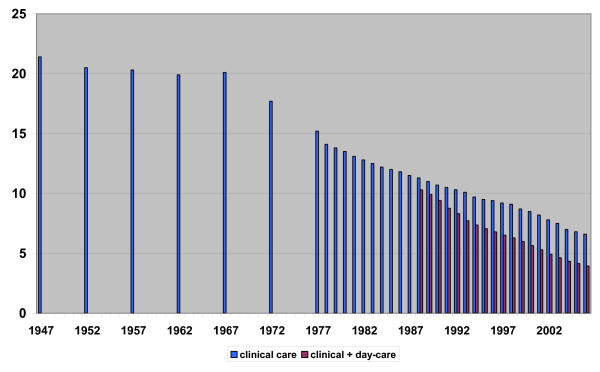
**Average length of stay in Dutch hospitals 'clinical care' and 'clinical + day-care'**. Source: 1947–1977 in 5-year intervals by CBS; 1978–2006 yearly data by LMR Prismant.

The length of stay in Dutch hospitals has been decreasing nearly every year since data have become available. In 1978 (which is the first year for which data from the LMR could be used) patients stayed in hospital for an average of 14.1 days, while in 2006 the average length of stay was reduced to only 6.6 days. This amounted to an average decrease of 0.3 days per year. In Figure 2 we have also plotted 5-year interval data made available by the CBS. This information dates back to 1947 when the average length of stay was 21.4 hospital days [8].

#### Variation in length of stay between hospitals

In 2000, the shortest average length of stay was 5.7 days while the longest was 11.3 days. The 15^th ^percentile hospital had an average length of stay of 7.4 days.

In 2006 more than 80% of all hospitals reached an average length of stay shorter than the 15^th ^percentile hospital in the year 2000. Between 2000 and 2006 the 15^th ^percentile decreased from 7.4 to 5.7 hospital days. The difference between the longest length of stay and the shortest length of stay also declined during this period: In 2000, the longest length of stay (11.3 days) was 2.0 times longer than the shortest length of stay (5.7 days), while in 2006 it was 1.7 times as long (longest 8.7 days and shortest 5.1 days).

Substantial variation in length of stay among hospitals will occur because not all hospitals have the same specialty (to the same extent) and also within a specialty hospitals can have a different patient mix.

**Figure 3:**
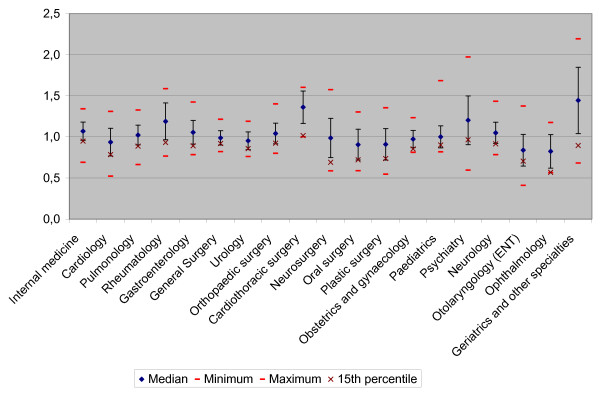
Variation in average length of stay for separate specialties, 2006.

Figure 3 shows the variation in average length of stay for the separate specialties in 2006. For each specialty the national range is identified from hospital-scores of the quotient of the actual length of stay and the expected length of stay. The figure shows that the greatest range of lengths of stay can be found in geriatrics and other specialties and psychiatry."

### 2. Potential reduction of hospital days in Dutch hospitals

In Table 1 we show the percentage of hospital days that could have been saved if all hospitals had substituted their potential day-care patients to day-care and treated their patients as efficiently as the 15th percentile hospital. This saving is expressed as a percentage of the total number of admissions in clinical and day-care.

**Table 1:** Percentage of hospital days that could have been saved

	% hospital days (clinical and day care) to gain by substitution to day care	% hospital days (clinical and day care) to gain by reduction length of stay to 15th percentile hospital	% hospital days (clinical and day care) to gain by substitution to day care AND reduction length of stay to 15th percentile hospital	Extrapolation to all Dutch hospitals: number of hospital days to gain
Internal medicine	0.3%	11.1%	11.4%	248231
Cardiology	1.2%	16.5%	17.7%	243766
Pulmonology	0.2%	12.9%	13.1%	114951
Rheumatology	0.1%	17.3%	17.4%	14357
Gastroenterology	1.4%	11.5%	12.9%	51784
General Surgery	2.5%	9.1%	11.6%	243697
Urology	4.7%	9.8%	14.5%	60074
Orthopaedic surgery	2.6%	10.7%	13.3%	127051
Cardiothoracic Surgery	0.0%	22.2%	22.2%	34833
Neurosurgery	0.4%	26.9%	27.3%	48463
Oral Surgery	3.2%	15.8%	18.9%	8712
Plastic surgery	4.1%	14.1%	18.2%	28022
Obstetrics and gynaecology	0.5%	11.5%	12.0%	126912
Paediatrics	0.2%	11.4%	11.6%	100307
Psychiatry	0.0%	19.1%	19.1%	84182
Neurology	0.1%	11.8%	11.9%	106441
Otolaryngology (ENT)	13.2%	10.5%	23.7%	72756
Ophthalmology	5.5%	13.9%	19.4%	37975
Geriatrics and other specialties	0.2%	38.7%	38.9%	71924

**TOTAL**	**1.4%**	**12.9%**	**14.3%**	**1824441**

In the last column of Table 1, we have calculated the total potential reduction of hospital days by applying the percentages of column 3 (Percentage hospital days to gain by substitution to day care **and **reduction length of stay to 15^th ^percentile hospital) to all hospital days in all Dutch hospitals.

Expressed in absolute numbers Internal Medicine is the specialty that has the largest number of hospital days to save, but expressed in percentages this potential reduction is the smallest. The standard deviation of the mean length of stay for Internal Medicine is relatively small when adjusted for case-mix (0.11). Therefore, the potential percentage reduction generated by reducing lengths of stay to the 15^th ^percentile hospital is relatively small, but because Internal Medicine is the largest specialty (in number of admissions), the absolute number of hospital days that can be saved is the highest of all specialties.

For General Surgery, the second largest specialty in the Netherlands, the data are similar. The standard deviation for General Surgery is the smallest of all specialties (0.09). The percentage of hospital days that could be saved is 11.6%. In comparison with Internal Medicine a larger portion of days could be gained by substitution to day-care.

'Geriatrics and other specialties' has the largest percentage of hospital days that could be saved by reducing length of stay to the 15^th ^percentile. The standard deviation is 0.40. This specialty mostly treats older multi-problem patients with multiple secondary diagnoses. They often are in need of long-term care in a nursing home or the community and may block hospital beds. They cannot leave the hospital in case of lacking nursing home capacity, insufficient home care arrangements or slow referral procedures. The differences in lengths of stay between hospitals that do not have problems in transferring these patients to long-term care facilities and hospitals that do have these problems are substantial.

Overall the average length of stay in Dutch hospitals – if all hospitals would be able to treat their patients like the 15^th ^percentile hospital – would be 6.0 days and day-care (that is not included in this length of stay) would grow by 13%.

## Discussion

### Implications for policy and practice

The continuous reduction of length of stay is all the more remarkable considering two main developments with an increasing effect on the average clinical length of stay:

1. Since the eighties of the last century many hospitals have introduced day-care and have increasingly substituted (short-term) clinical admissions for day-care [9,10].

2. Another development which had an increasing effect on the average length of stay is the ageing of the patient population. In 1978, 19% of the admissions were 65 years or older. In 2006, this increased to 48%. On average, elderly people stay longer in hospitals than younger ones; in 2006 the 0–64-year-old patient stayed an average 5.2 days in hospital and the patients aged more than 64 years stayed an average of 9.1 days.

In spite of these two developments the average length of stay decreased from year to year. We expect this to continue because in the coming years, the financing system in Dutch hospitals will more and more be based on market forces and the reimbursement through payments per diem will be abolished (as in the United States more than two decades ago [11]). The increased competition among hospitals will increase interest in length of stay reduction in order to increase capacity for additional admissions and improve financial performance.

### Limitations of the study

#### Chance of underestimation

The potential reduction in length of stay may in fact be higher because of two methodological choices.

First, we have chosen to use a 1997 list of treatments that could have been performed in day care. This list could have been longer if we had used more recent data as a reference. Currently, we are planning to update the list. Probably a new list will show more possibilities to substitute inpatient care into day-care. Until now, the health care system in the Netherlands gave only few incentives to treat patients in day-care. Updating the list at this moment will also give an underestimation of the possibilities for day-care. We think that, when the changes in the financing system have been carried out entirely, an update will clearly show more possibilities for day-care.

Second, in our standardisation for patient mix, the expected length of stay was not used for patients with a length of stay of 100 hospital days and longer and for patients who died in hospital. For these two groups the realised length of stay was used instead of the expected length of stay. This means that the results are without the potential gain in efficiency for these two groups. However, it concerns a small number of patients. Only 0.1% of all patients had a length of stay of 100 hospital days and longer and 2.4% of all patients died in hospital.

#### Specialty as a variable for length of stay

The variation in the quotients of actual length of stay and expected length of stay shows that for several specialties the mean score is not 1. This is the case especially for cardiothoracic surgery and for 'other specialties'. For these two specialties it is 'normal' that the quotient of actual and expected length of stay is higher than 1.0. For 'other specialties' it is known that many hospitals created a special ward for patients that could not be discharged in time to next care facilities like nursing homes. The length of stay of these patients was longer because of these waiting days and the hospitals booked for these patients an administrative transfer to 'other specialties'. The code 'other specialties' is also used for geriatrics. This specialty treats patients that may have the same age group, diagnosis- and procedure group as patients treated by other specialty, but often the patients treated by geriatrics have a more complex syndrome and stay longer in hospital because of their frailty. The variables for standardisation (age group, diagnosis- and procedure group) do not seem to be sufficient for patients that are discharged by these two specialties. The variable 'specialty' should also been taken into account. Because we did our analysis for each separate specialty this was no problem for this study, but if length of stay is benchmarked on the level of hospitals, 'specialty' is a variable that should be taken into account.

#### Lack of data based on severity of illness

For a large part of the data, adjustment for age, primary diagnosis and procedure amounts to an adjustment for severity of illness. However, we realise that there may still be residual case-mix related variation that is not adjusted for. We did not adjust for variations in comorbidities Neither did we account for variations between elective versus emergency cases. Both parameters were recorded in the LMR, but the completeness of the registration of these items varies between hospitals. We realise that the presence or absence of a large number of comorbidities and/or emergency cases at hospital level will affect overall length of stay of a particular hospital. However, this potential residual variation that is not adjusted for is one of the reasons why we used the 15^th ^percentile as benchmark and not the minimum. If a more sophisticated comparison data based on severity of illness were available, it would be possible to identify which subpopulations (younger, older, diagnosis, procedure, long stay, short stay) were generating the largest numbers of excess days. This could be possible in the future because the Dutch hospital information system will be upgraded in 2010.

### Perspectives for future research

Length of stay is often used as an indicator of efficiency [6,11-13]. Efficiency can be described as the relationship between input and output. From a hospital perspective a length of stay reduction may increase efficiency by increasing the output (number of patients) or decreasing the inputs (e.g. available beds for inpatient care). Both may be realised by reducing 'waiting'-days during a hospital stay or by minimising time between examinations, consultations and procedures. However, if the reduction in lengths of stay results in increased intensity of care (and consequently cost) the efficiency improvement may be smaller. In addition, the reduction of hospital days will mainly be a reduction of 'low care' days. The more intensive and expensive patients remain in the hospital.

From a health system perspective, efficiency also depends on the efficiency of other sectors and on health outcomes [14]. When length of stay reduction is realised by a quicker transfer to follow-up care, the costs of care may be passed. Quicker discharge may increase the pressure on other health care sectors (and their cost) and as a result, the efficiency of the health care system may not improve. Therefore, more insight into the relationship between length of stay and quality of care in the hospital is needed [15-17]. Shorter lengths of stay may also lead to a better quality of care, and, conversely, a better quality of care can lead to a shorter length of stay. For example fewer hospitals days will reduce the chance for complications such as infections and fewer complications will lead to shorter lengths of stay.

On the contrary, we did not find research that showed that shorter lengths of stay in hospitals is related to adverse quality [15,18,1,5]. Only for some specific procedures or diagnoses there is information concerning the limits of hospital stay reduction [19].

Brownell stated that 'reassuringly, shorter stays have not been found to be related to adverse patient outcomes. In fact, a study of almost 4000 US hospitals showed that hospitals that discharged patients more efficiently had lower post discharge death rates' [5]. Finally, Harrison observed: 'Improving hospital efficiency by shortening length of stay does not appear to result in increased rates of readmission or numbers of physician visits within 30 days after discharge from hospital. Research is needed to identify optimal lengths of stay and expected readmission rates' [16].

If quality improvement leads to shorter lengths of stay and shorter lengths of stay can lead to a better quality of care, we are curious if hospitals with shorter length of stay have better outcomes than hospitals with a longer length of stay. In future work we will investigate the connection between length of stay and quality of care.

## Conclusion

The length of stay in Dutch hospitals has been decreasing for decades. Between 1978 and 2006 the average decrease was 0.3 days per year. In 2006 more than 80% of all hospitals reached an average length of stay lower than the 15th percentile hospital in the year 2000. In 2006 the length of stay ranged from 5.1 to 8.7 among the 69 hospitals. Still, a further reduction of lengths of stay is possible. If all hospitals had substituted their potential day-care patients to day-care and if the average length of stay of the 15^th ^percentile hospital in 2006 is taken as the standard, a 14% reduction of all hospital days would be attained. This percentage varied substantially across medical specialties (e.g. internal medicine 11% and ENT specialty 24%). Extrapolating the potential reduction of lengths of stay of the 69 hospitals (that participate in the LMR) to all 98 Dutch hospitals yields a total reduction of 1.8 million hospital days.

## Competing interests

The authors declare that they have no competing interests.

## Authors' contributions

IB designed the study, performed the analysis, interpreted the results, and drafted the manuscript. RH, TK and RJL helped to interpret the results and contributed to the discussion GPW supervised the study and participated in the formulation of the discussion. All authors reviewed and edited the manuscript for intellectual content.

## Pre-publication history

The pre-publication history for this paper can be accessed here:


